# A 10-Year Trend Analysis of Heart Failure in the Less Developed Brazil

**DOI:** 10.36660/abc.20180321

**Published:** 2020-02

**Authors:** Amanda D. F. Fernandes, Gilson C. Fernandes, Manuel Rivera Mazza, Leonardo M. Knijnik, Gustavo Soares Fernandes, Andre Telis de Vilela, Amit Badiye, Sandra V. Chaparro

**Affiliations:** 1 University of Miami Miller School of Medicine - Department of Medicine, Miami, Florida - USA; 2 University of Miami Miller School of Medicine - Division of Cardiology, Miami, Florida - USA; 3 Centro Universitario de Joao Pessoa, João Pessoa, PB - Brazil; 4 Universidade Federal da Paraiba, João Pessoa, PB - Brazil

**Keywords:** Heart Failure/physiopathology, Heart Failure/mortality, Heart Failure/epidemiology, Comorbidity, Heart Failure/trends, Hospitalization

## Abstract

**Background:**

Data on heart failure (HF) epidemiology in less developed areas of Brazil are scarce.

**Objective:**

Our aim was to determine the HF morbidity and mortality in Paraiba and Brazil and its 10-year trends.

**Methods:**

A retrospective search was conducted from 2008 to 2017 using the DATASUS database and included patients ≥ 15 years old with a primary diagnosis of HF. Data on in-hospital and population morbidity and mortality were collected and stratified by year, gender and age. Pearson correlation and linear-by-linear association test for trends were calculated, with a level of significance of 5%.

**Results:**

From 2008 to 2017, HF admissions decreased 62% (p = 0.004) in Paraiba and 34% (p = 0.004) in Brazil. The in-hospital mortality rate increased in Paraiba and Brazil [65.1% (p = 0.006) and 30.1% (p = 0.003), respectively], but the absolute in-hospital mortality had a significant decrease only in Paraiba [37.5% (p = 0.013)], which was maintained after age stratification, except for groups 15-19, 60-69 and > 80 years. It was observed an increase in the hospital stay [44% (p = 0.004) in Paraiba and 12.3% (p = 0.004) in Brazil]. From 2008 to 2015, mortality rate for HF in the population decreased 10.7% (p = 0.047) in Paraiba and 7.7% (p = 0.017) in Brazil.

**Conclusions:**

Although HF mortality rate has been decreasing in Paraiba and Brazil, an increase in the in-hospital mortality rate and length of stay for HF has been observed. Hospital-based clinical studies should be performed to identify the causes for these trends of increase.

## Introduction

Heart Failure (HF) is the main cause of hospitalizations in the United States in patients older than 65 years old,^1,2^ and is estimated to affect 26 million people worldwide.^3^ Its prevalence has been increasing fast due to aging of the population.^1,4^ A higher life expectancy has been achieved with adherence to medical therapy, ventricular assist devices (VADs) and increase in the number of heart transplants.^1^

Paraiba is one of the nine states of the Northeast region of Brazil and had an estimated population in 2017 of 4,025,558 inhabitants, corresponding to the 13^th^ highest population among the 27 federative units of Brazil. The gross domestic product per capita of Paraiba was US$3,594.94 in 2010, corresponding to fourth poorest state in the country, and the human development index in 2014 was 0.701, the 6^th^ lowest in the country.^5,6^ Data regarding the epidemiology of HF in less developed countries are still limited and based mainly in cohorts of hospitalized patients or clinical trials.^2^ In Brazil, there is no data about the epidemiology of HF in Paraiba, and only a few reports on HF statistics in the Northeast region of Brazil.^7,8^

A better understanding of the HF epidemiology in less developed areas of Brazil, as Paraiba, through a population-based study, could lead to a more effective and appropriate healthcare planning. The aim of this study was to describe and to perform a 10-year trend analysis of the HF morbidity and mortality in the state of Paraiba and in Brazil.

## Methods

### Study model

This is a population-based time series analysis using the Hospital Information System (SIH/SUS), available at DATASUS (Department of Informatics of the Brazilian Unified Health System- SUS) database.^6^ DATASUS is responsible for the administration of health and financial information declared by all states and cities, and the federal district of Brazil. This database compiles information regarding health assistance, epidemiology, morbidity and demography.

### Study population

The population of interest was composed by Brazilians older than 15 years that used any healthcare services under the primary diagnosis of HF, represented by the code I50 of the International Classification of Diseases 10^th^ Revision (ICD-10), between 2008 and 2017.

### Variables

Epidemiological data on HF were extracted, including absolute and relative mortality of the population, in-hospital mortality (absolute numbers), in-hospital mortality rate, number of hospital admissions and length of hospitalization. Variables were stratified by year, gender and age groups (15-19, 20-29, 30-39, 40-49, 50-59, 60-69, 70-79 and ≥ 80 years). In-hospital data from the period of 2008 to 2017, and population data from 2008 to 2015 were available. The last population census conducted by the Brazilian Institute of Geography and Statistics (IBGE)^5^ in 2010 was also used.

### Data analysis

Categorical variables were expressed as frequencies and continuous variables as mean ± standard deviation (SD).

In-hospital mortality rate from HF was obtained by dividing the number of all in-hospital HF deaths in Paraiba or Brazil by the number of hospitalizations for HF in the corresponding year. Population mortality rate from HF was calculated by dividing the number of all HF deaths in Paraiba or Brazil by the respective population in the corresponding year.

The Statistical Package for the Social Sciences (SPSS) version 21.0 (SPPS Inc., Chicago, USA) was used for the analysis. We used the Shapiro-Wilk’s test to test the normality of data distribution for further analyses. The Pearson correlation was used to evaluate the correlation between numerical variables with normal distribution. The Chi-square test was performed using a contingency table and the linear-by-linear association test, also known as Mantel-Haenzsel test for trends, which is equivalent to the Cochran-Armitage test for trends available in other statistical packages.^9^ The level of significance was set at 5%.

## Results

Descriptive statistics of our variables are presented in Table 1.

**Table 1:** Descriptive statistics of Heart Failure epidemiology in Paraiba, from 2008 to 2017

Variables	Mean	Standard deviation	Shapiro-Wilk significance*
Deaths (population)^†^	739.88	32.92	0.385
Women	387.88	24.07	0.916
Men	349.63	17.71	0.099
Deaths (in-hospital)	474.50	95.52	0.324
Women	238.80	46.15	0.763
Men	234.70	52.04	0.161
Population mortality rate (per 100,000) ^†^	19.16	1.09	0.775
In-hospital mortality rate (per 100)	9.76	1.84	0.659
Number of admissions	5117.20	1805.13	0.176
Mean duration of hospitalization (days)	5.92	0.80	0.121

* In the Shapiro-Wilk test, the null hypothesis is that the population follows a normal distribution; if p < 0.05, the data is not normally distributed;

† Year interval: 2008-2015;

Source of data: SUS Information System (DATASUS) and Brazilian Institute of Geography and Statistics (2010).

### Hospitalizations

The total number of HF admissions in Paraiba state between 2008 and 2017 was 51,172, representing the leading cause of hospitalizations due to cardiovascular diseases (29.4%), followed by other ischemic diseases of the heart (13%), stroke (11%), primary hypertension (10%) and acute myocardial infarction (5%). During the same period, HF was also the leading cardiovascular cause of hospitalization in Brazil, with 2,380,133 cases (21%). HF was responsible for 2.54% and 2.25% of all causes of hospitalization in Paraiba and in Brazil, respectively.

**Figure 1:**
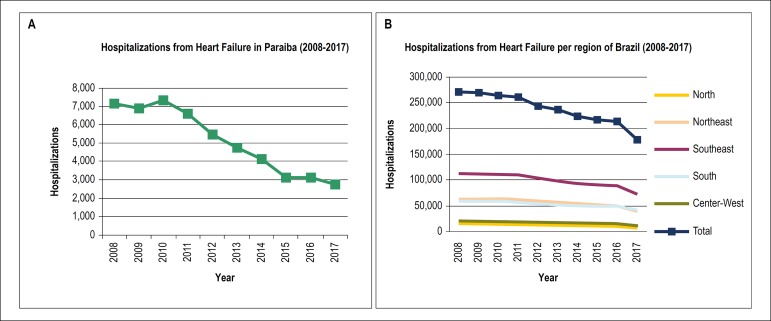
Trends in absolute number of hospitalizations from heart failure from 2008 to 2017 in Paraiba (A) and regions of Brazil (B).

A downward trend in the absolute number of hospitalizations from HF in Paraiba and Brazil was observed between 2008 and 2017, corresponding to a decrease of 62% (R = -0.970; p = 0.004; Table 2; Figure 1A) and 34% (R = -0.964; p = 0.004; Table 3; Figure 1B), respectively. The frequency of males hospitalized for HF was 52% in Paraiba and 51% in Brazil.

**Table 2:** Heart failure trends in Paraiba, from 2008 to 2017

Variables	2008	2009	2010	2011	2012	2013	2014	2015	2016	2017	P values for trends*^†^	Pearson (R)
Number of deaths (population)	722	777	772	753	719	768	725	683	------	------	0.175	-0.513
Women	365	398	390	413	386	421	383	347	------	------	0.665	-0.164
Men	351	374	379	337	332	347	341	336	------	------	0.130	-0.573
Number of deaths (in-hospital)	472	588	581	561	551	462	461	374	378	317	0.013*	-0.824
Women	220	310	294	282	265	235	224	207	188	173	0.022*	-0.762
Men	252	278	287	279	286	227	237	167	190	144	0.012*	-0.837
Mortality rate (population)	19,25	20.53	20.21	19.55	18.51	19.62	18.38	17.19	------	------	0.047*	-0.751
Mortality rate (in-hospital)	6.60	8.50	7.90	8.50	10.10	9.8	11.20	12.00	12.10	10.90	0.006*	0.917
Women	6.35	9.32	8.52	9.02	10.08	10.39	11.49	13.56	12.69	11.93	0.006*	0.908
Men	6.76	7.67	7.36	8.15	10.20	9.12	11.02	10.61	11.59	9.40	0.013*	0.828
Number of admissions	7143	6890	7331	6571	5450	4739	4102	3112	3115	2719	0.004*	-0.970
**Deaths per age range (population)**												
15-19 years	2	2	3	2	1	0	1	1	------	------	0.067	-0.693
20-29 years	3	8	8	5	5	6	2	6	------	------	0.588	-0.205
30-39 years	8	16	9	13	9	10	10	8	------	------	0.389	-0.326
40-49 years	21	22	29	23	25	23	24	13	------	------	0.292	-0.399
50-59 years	47	42	53	33	45	48	46	36	------	------	0.479	-0.267
60-69 years	84	88	92	75	92	101	82	93	------	------	0.457	0.281
70-79 years	164	172	152	172	147	179	174	154	------	------	0.979	-0.010
Older than 80 years	387	422	423	427	394	401	385	372	------	------	0.143	-0.553
**Deaths per age range (in-hospital)**												
15-19 years	3	2	3	3	2	2	2	2	4	2	0.815	-0.078
20-29 years	11	10	8	5	5	4	4	7	2	2	0.010*	-0.859
30-39 years	17	19	16	11	16	12	7	8	6	8	0.008*	-0.887
40-49 years	24	35	35	35	36	30	19	10	10	17	0.029*	-0.727
50-59 years	55	53	53	55	63	47	46	24	38	35	0.025*	-0.748
60-69 years	77	97	96	97	101	86	93	72	76	44	0.061	-0.625
70-79 years	129	150	136	142	131	119	129	96	89	75	0.009*	-0.865
Older than 80 years	149	213	227	211	194	157	156	153	148	129	0.052	-0.649
Mean duration of hospitalization (days)	5	5.2	5.3	5.5	5.6	5.6	6.1	6.5	7.2	7.2	0.004*	0.953

* p < 0.05;

† P value for trends according to the linear-by-linear association;

Source of data: SUS Information System (DATASUS) and Brazilian Institute of Geography and Statistics (count of 2010).

**Table 3:** Heart failure trends in Brazil, from 2008 to 2017

Variables	2008	2009	2010	2011	2012	2013	2014	2015	2016	2017	P values for trends*^†^	Pearson (R)
Number of deaths (population)	27,567	27,314	27,544	27,818	26,694	27,290	26,783	27,434	------	------	0.276	-0.412
Women	13,990	14,136	14,236	14,525	13,824	14,014	13,846	14,435	------	------	0.929	0.034
Men	13,428	13,047	13,159	13,130	12,756	13,166	12,825	12,900	------	------	0.069	-0.689
Number of deaths (in-hospital)	22,513	23,043	23,667	24,451	23,071	22,858	22,031	22,756	23,519	19,209	0.131	-0.504
Women	11,021	11,356	11,740	12,099	11,426	11,305	10,963	11,450	11,738	10,509	0.401	-0.280
Men	11,198	11,395	11,676	12,092	11,408	11,301	10,823	11,097	11,577	10,213	0.119	-0.520
Mortality rate (population)	14.54	14.26	14.44	14.46	13.76	13.57	13.21	13.42	------	------	0.017*	-0.905
Mortality rate (in-hospital)	8.3	8.5	8.9	9.4	9.5	9.7	9.8	10.5	11.0	10.8	0.003*	0.981
Women	8.5	8.7	9.2	9.6	9.8	10.0	10.2	11.0	11.5	11.2	0.003*	0.980
Men	8.2	8.4	8.7	9.2	9.3	9.4	9.6	10.1	10.6	10.4	0.003*	0.985
Number of admissions	270,988	269,891	265,038	260,995	242,919	236,550	223,825	217,050	214,432	178,445	0.004*	-0.964
**Deaths per age range (population)**												
15-19 years	56	55	47	49	51	41	40	35	------	------	0.014*	-0.926
20-29 years	206	204	180	176	165	174	159	145	------	------	0.012*	-0.947
30-39 years	468	415	379	387	396	373	358	355	------	------	0.022*	-0.863
40-49 years	1,039	1,002	1,034	957	913	920	836	815	------	------	0.011*	-0.957
50-59 years	2,389	2,303	2,293	2,269	2,259	2,214	2,072	2,157	------	------	0.016*	-0.910
60-69 years	4,296	4,249	4,196	4,268	4,057	4,230	4,123	4,255	------	------	0.328	-0.370
70-79 years	7,178	7,027	7,062	7,013	6,727	6,969	6,707	6,845	------	------	0.037*	-0.788
Older than 80 years	11,788	11,928	12,206	12,539	12,012	12,259	12,383	12,733	------	------	0.039*	0.782
**Deaths per age range (in-hospital)**												
15-19 years	87	97	75	73	61	65	59	61	61	38	0.007*	-0.898
20-29 years	284	271	264	241	220	230	188	176	175	152	0.003*	-0.984
30-39 years	597	529	503	501	475	451	445	437	434	369	0.008*	-0.887
40-49 years	1,278	1,216	1,227	1,216	1,104	1,102	981	1,004	1,052	853	0.005*	-0.931
50-59 years	2,687	2,722	2,736	2,793	2,700	2,620	2,372	2,498	2,569	2,149	0.019*	-0.783
60-69 years	4,358	4,578	4,739	4,769	4,599	4,452	4,439	4,601	4,783	4,149	0.533	-0.208
70-79 years	6,337	6,371	6,583	6,820	6,237	6,343	5,984	6,174	6,496	5,706	0.101	-0.546
Older than 80 years	6,591	6,967	7,289	7,778	7,438	7,343	7,318	7,596	7,745	7,306	0.064	0.617
Mean duration of hospitalization (days)	6.5	6.4	6.5	6.6	6.7	6.9	7.1	7.3	7.4	7.3	0.004*	0.960

* p < 0.05;

† P value for trends according to the linear-by-linear association;

Source of data: SUS Information System (DATASUS) and Brazilian Institute of Geography and Statistics (count of 2010)

When stratified by age, individuals older than 60 years old corresponded to 71% and 73% of all the cases of HF admissions in Paraiba and Brazil, respectively, with the highest frequency in the age range from 70 to 79 years old.

### Absolute mortality of population

The absolute mortality from HF of the population showed a non-significant decline from 2008 to 2015 in Paraiba (R = -0.513; p = 0.175; Table 2) and Brazil (R = -0.412; p = 0.276; Table 3), with no difference by gender. Women represented 53% of deaths in Paraiba and 52% in Brazil. In Paraiba, the decrease in absolute deaths from HF in the population across all age categories was not statistically significant (Table 2).

Between 2008 and 2015, the highest proportion of deaths from HF occurred at the age group of ≥ 80 years old in both men and women in Paraiba (50% and 59%, respectively) and in Brazil (38% and 52%, respectively). The proportions of deaths from HF at the age ≥ 60 years old in Paraiba was 87% in men and 90% in women and, in Brazil, 83% in men and 89% in women.

### Population mortality rate

**Figure 2:**
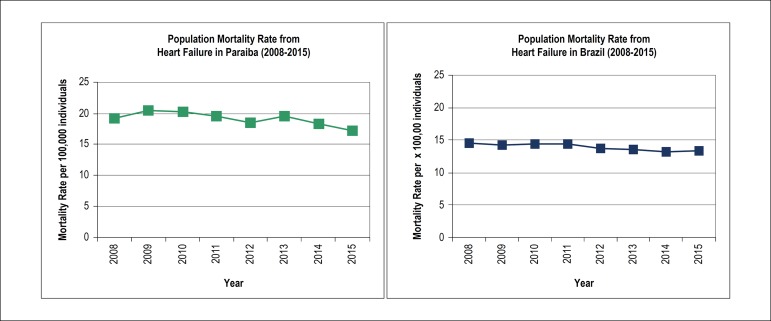
Trends in population mortality rate (per 100,000 inhabitants) from heart failure in Paraiba (green) and Brazil (blue) from 2008 to 2015.

The mean mortality rate from HF in the population was 19.2/100,000 (±1.09) in Paraiba and 14.0/100,000 (±0.53) in Brazil, with a significant decline of 10.7% (R = -0.751; p = 0.047; Table 2) in Paraiba and 7.7% (R = -0.905; p = 0.017; Table 3) in Brazil between 2008 and 2015, respectively (Figure 2).

### Absolute in-hospital mortality

**Figure 3:**
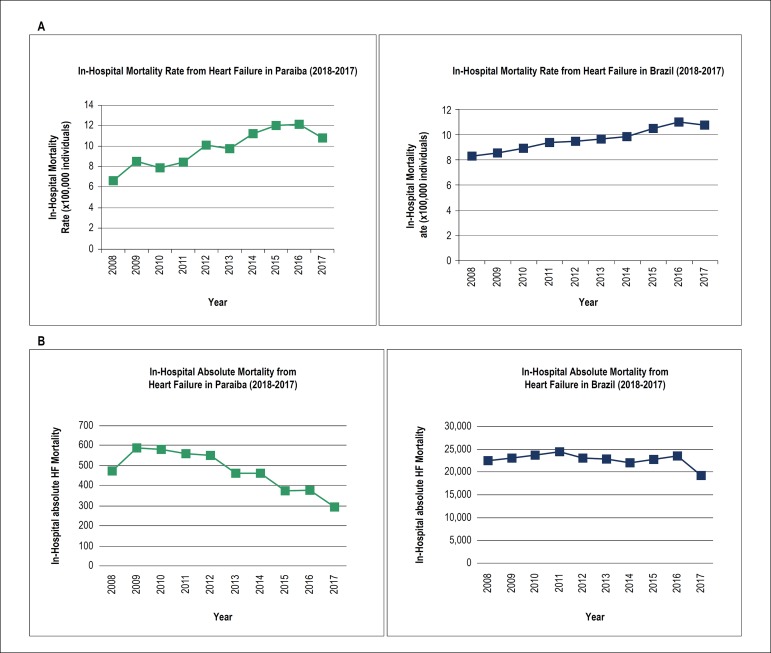
(A) Trend of the in-hospital mortality rate from heart failure in Paraiba (green) and Brazil (blue) from 2008 to 2017; (B) trend of the in-hospital absolute mortality from heart failure in Paraiba (green) and Brazil (blue) from 2008 to 2017.

The absolute in-hospital HF mortality, between 2008 and 2017, showed a significant decrease of 37.5% in Paraiba (R = -0.824; p = 0.013; Table 2; Figure 3B) and a non-significant 14.6% decrease in Brazil (R = -0.504; p = 0.131; Table 3; Figure 3B). In the stratified analysis, a significant decrease in the absolute in-hospital deaths from HF was observed for both men and women in Paraiba (R = -0.837; p = 0.012 and R = -0.762; p = 0.022; Table 2); this statistically significant trend by sex was not observed in Brazil (Table 3).

**Figure 4:**
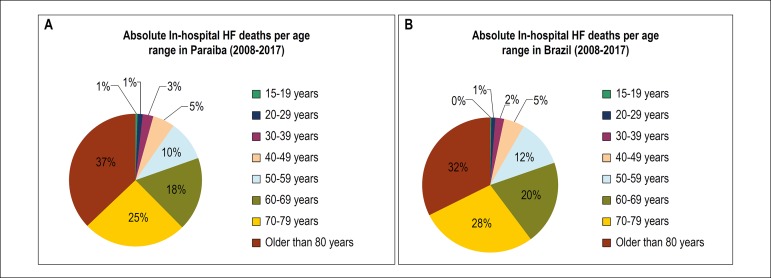
(A) Absolute in-hospital deaths from heart failure in Paraiba per age range, from 2008 to 2017. (B) Absolute in-hospital deaths from heart failure in Brazil per age range, from 2008 to 2017.

Individuals older than 80 years old presented the highest proportion of absolute in-hospital HF deaths in Paraiba and Brazil, from 2008 to 2017, (37% and 32%, respectively) (Figure 4). In Paraiba, there was a statistically significant reduction in in-hospital deaths from HF for the age categories: 20-29 years (p = 0.010), 30-39 years (p = 0.008), 40-49 years (p = 0.029), 50-59 years (p = 0.025) and 70-79 years (p = 0.009) (Table 2).

Further data on the absolute number of in-hospital deaths from HF per age range in Brazil are specified in Table 3.

### In-hospital mortality rate

The in-hospital HF mortality rate increased significantly by 65.1% in Paraiba (R = 0.917; p = 0.006; Table 2), from 6.6% in 2008 to 10.9% in 2017, and by 30.1% in Brazil (R = 0.981; p = 0.003; Table 3), from 8.3% in 2008 to 10.8% in 2017 (Figure 3A). The increase in in-hospital mortality rate from HF by gender was also significant for both men and women in Paraiba (R = 0.828; p = 0.013 and R = 0.908; p = 0.006, respectively; Table 2). This trend was also observed for both sex in Brazil, in a similar magnitude of effect (R = 0.985; R = 0.980; p = 0.003; Table 3).

**Figure 5:**
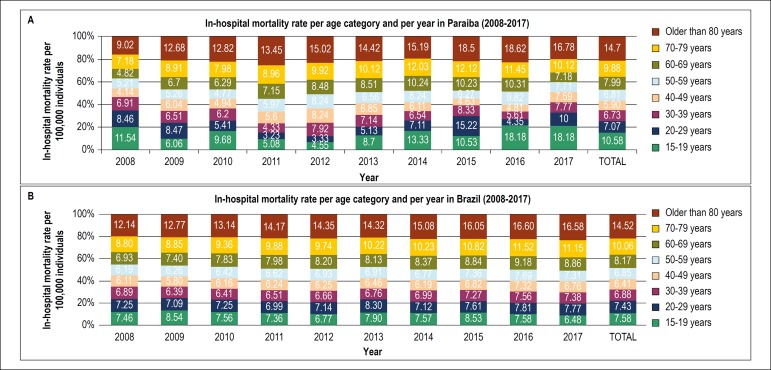
(A) Trend of the in-hospital mortality rate from heart failure in Paraiba per year and age range, from 2008 to 2017. (B) Trend of the in-hospital mortality rate from heart failure in Brazil per year and age range, from 2008 to 2017.

The in-hospital HF mortality rate per age range was highest in individuals older than 80 years old, with a mean of 14.7% in Paraiba and 14.5% in Brazil (Figure 5) from 2008 to 2017. In this age range, the in-hospital mortality rate from HF per gender in Paraiba was 12.4% in men and 15.2% in women, and in Brazil, 13.7% in men and 14.9% in women.

### Length of hospital stay

**Figure 6:**
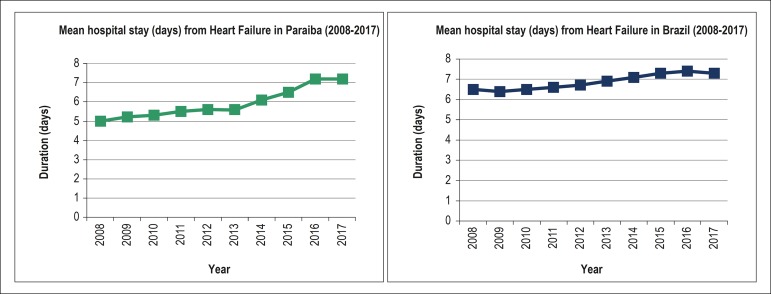
Trends in the mean length of stay (days) from heart failure hospitalizations in Paraiba (green) and Brazil (blue) from 2008 to 2017

The average length of hospital stay for HF was 5.9 days (±0.8) in Paraiba and 6.9 days (±0.4) in Brazil, with a significant increase of 44% (R = 0.953; p = 0.004; Table 2) and 12.3% (R = 0.960; p = 0.004; Table 3), respectively, between 2008 and 2017 (Figure 6). In Table 4, we present the duration of hospital stay per year, and the associated cost, both in Paraiba and Brazil.

**Table 4:** Total cost of HF hospitalizations (US$) and duration of HF admission (days) in Paraiba and Brazil, from 2008 to 2017

	PARAÍBA		BRAZIL	
Year	Total cost with hospitalizations (US$)	Duration of admission (days)	Total cost with hospitalizations (US$)	Duration of admission (days)
2008	1,762,825.91	5	77,940,473.93	6.5
2009	2,286,531.90	5.2	89,837,575.25	6.4
2010	2,541,429.71	5.3	92,835,802.31	6.5
2011	2,378,139.40	5.5	93,939,042.90	6.6
2012	1,939,284.53	5.6	91,509,632.22	6.7
2013	1,694,005.09	5.6	93,561,446.18	6.9
2014	1,578,506.24	6.1	96,199,113.56	7.1
2015	1,233,302.85	6.5	99,069,494.68	7.3
2016	1,249,580.27	7.2	102,181,019.88	7.4
2017	1,103,600.05	7.2	85,390,241.41	7.3
Total	17,767,205.95	5.9 ± 0.8 (mean ± SD)	922,463,842.32	6.9 ± 0.4 (mean ± SD)

SD: standard deviation;

Source of data: SUS Information System (DATASUS) and Brazilian Institute of Geography and Statistics (count of 2010)

## Discussion

To our knowledge, this is the first study to describe the trends of HF epidemiology in a less developed region of Brazil. Information regarding the incidence, prevalence, morbidity and mortality of HF in Latin America and the Caribbean (LAC) are heterogeneous and scarce. Most of the data come from South America (92%), with 86% of the studies conducted in Brazil and Argentina.^10^ In Brazil, most of the published data come from developed areas, the Southeast and South regions.

HF was the leading cause of hospitalizations among cardiovascular diseases in Paraiba and Brazil, and corresponded to 2.54% and 2.25% of all admissions, respectively. Similarly, in the U.S., HF was the cause of more than 1 million admissions per year from 2001 to 2009,^11^ and represented 1-2% of all hospitalizations.^3^

Fang et al.^12^ performed a study to determine the trends in HF in the U.S. using the National Hospital Discharge Survey data from 1979 to 2004, and observed an increase of 185% in the absolute number of HF admissions (from 409,000 to 1,166,000) and the HF hospitalization rates (per 100,000) increased from 219 to 390 during the same period. Other authors, however, have reported that the number of primary hospitalizations for HF have been decreasing in the U.S. between 1.0% to 4.3% per year since 2001.^11,13^ In the LAC, Godoy et al.^14^ showed a 32% decrease in HF admissions, between 1992-1993 and 2008-2009, which is consistent with our findings of a 34% decrease in the absolute number of hospitalizations for HF in Brazil, and 62% in Paraiba. This observed reduction can be a sign of improvement in the overall management of the risk factors for HF,^4^ a decrease in the incidence of ischemic heart disease,^15^ and an improvement in HF management.^16^

Hospitalizations for HF in Paraiba and Brazil were more common for individuals between the ages of 70 to 79 years old. Individuals older than 60 years old represented 71% and 73% of admissions for HF in Paraiba and Brazil, respectively; this is similar to the frequency (70%) reported in previous studies in LAC and U.S..^2,4^ In Paraiba and in Brazil, the proportion of women hospitalized for HF was 48% vs 49%, similar to studies in the U.S., with 40% to 50% of women.^3,17^ A small study performed in a community in Brazil reported that 58% of hospitalized patients with HF were women.^18^ Also, the I Brazilian Registry of Heart Failure (BREATHE registry)^8^ describes that 60% of 1,263 admissions for HF in 51 centers of Brazil were women.

In our study, mean mortality rate for HF in the population between 2008 to 2015 was 19.2/100,000 (±1.09) cases in Paraiba and 14.0/100,000 (±0.53) cases in Brazil, with a decline of 10.7% and 7.7%, respectively. A decrease in the mortality rate for HF was also reported in Brazil and Argentina: in Sao Paulo, Brazil’s largest city, there was a 29% decrease, from 19.1/100,000 (1992-1993) to 13.6/100,000 (2008-2009);^14^ in Argentina, a nationwide study showed a reduction of 23% in the population HF mortality rate from 1995 to 2005.^10,19^ In the U.S., Go et al.^20^ compared the absolute number of HF deaths from 1995 to 2010, and found a decrease of 2.8% (287,000 vs 279,000), which potentially represents a significant decrease in the mortality rate, given the increase in the US population over 15 years.

Our study reports a mean in-hospital mortality rate for HF in Paraiba of 9.2% between 2008 and 2017. A prospective study performed in 51 centers from all the Brazilian regions, only with patients hospitalized due to acute HF, reported a total of 12.6% deaths in 1,263 hospitalized patients.^8^ In the LAC, a meta-analysis of 37 studies revealed a similar in-hospital mortality of 11.7%.^10^

Our study demonstrated an increase in the in-hospital mortality rate for HF, both in Paraiba and in Brazil (65% and 30%, respectively), between 2008 and 2017. Godoy et al.,^14^ between 1992-1993 and 2008-2009, also reported a 15% increase in the previous 15% in-hospital mortality rate in Brazil. In the U.S., however, the in-hospital mortality rate decreased from 4.5% in 2001 to 2.9% in 2014 according to a study that included patients with a primary diagnosis of HF.^13^ The decrease in the number of hospitalizations for HF during the study period, both in Paraiba and Brazil, is the most likely reason for the increased in-hospital mortality rate. Another plausible explanation could be the increased survival of HF patients, leading to a higher number of elderly patients, with more advanced HF and multiple comorbidities, and increased risk of death during hospitalization. Lastly, it is important to consider the lack of advanced therapies in less developed areas, as mechanical devices and heart transplantation, contributing to this trend of increased HF mortality rate in Paraiba, Brazil and LAC.

Although there was an increase in the in-hospital mortality rate, absolute in-hospital mortality showed a significant decrease of 37.5% in Paraiba and 14.6% in Brazil for the same period. In the U.S., Bueno et al.^21^ also observed a 50% decrease in the in-hospital mortality for HF in a population of elderly Medicare patients, from 1993 to 2008, and Ni and Xu,^22^ a 30% decrease.

Women represented 53% and 52% of the absolute mortality for HF in Paraiba and Brazil, respectively. The in-hospital mortality for HF in Paraiba had a similar proportion of women (50.5%). In the U.S., in 2010, 54.6% of all HF deaths happened in women.^20^ Hsich et al.^23^ observed no difference in the in-hospital mortality between women and men considering both the reduced and preserved ejection fraction groups.

Between 2008 and 2017, the mean duration of hospitalization for HF was 5.9 (±0.8) days in Paraiba and 6.8 (±0.4) days in Brazil, with an increase of 44% and 12.3%, respectively. In the LAC, Bocchi et al.^(2, 24)^ found a mean hospital stay of 5.8 days between 1998 and 2012. Ciapponi et al.^10^ reported an average of 7 days in 18 studies, and Godoy et al.^14^ found an increase of 25% in the length of stay, from 8.8 (1992-1993) to 11.3 days (2008-2009) in Brazil. In the U.S., two authors reported a decrease in the length of stay due to HF, from 8.8 to 6.3 days (1993-2008)^21^ and from 6.8 days (1999-2000) to 6.4 days (2007-2008).^4^

In the U.S., the per capita cost with healthcare was greater than the per capita gross domestic product of Paraiba (US$8,364.00 and US$3,594.94, respectively).^24^ The lower socioeconomic status in Paraiba may represent a risk factor for the high morbidity and mortality observed in our study, because the population has limited access to effective HF treatment.^24^ In the U.S., 52.5% of people with a household income less than US$10,000 suffer from a cardiovascular disease^20,25^ and Eapen et al.^26^ found that a higher income was associated with lower odds of 30-day mortality after a HF admission.

### Limitations

This is a retrospective and observational study, and the lack of patient-level data limited our ability to establish relationship between variables. Since our data was derived from a national database, it is likely that underreporting and misreporting of data have occurred. Also, since readmissions are not considered in the total number of HF hospitalizations, in-hospital mortality rate may have been underestimated.

## Conclusions

This is the first study to analyze the epidemiology of HF in Paraiba, a less developed state of Brazil, and to compare the results with national and international data. Over the last 10 years, the increase of the in-hospital mortality rate for HF in Paraiba and in Brazil followed the LAC trend, whereas the increase in the duration of hospitalization for HF is opposite to the decrease seen in the U.S.. In Paraiba and Brazil, we observed a decrease in admission for HF as primary diagnosis as well as in the absolute in-hospital deaths for HF, agreeing with the LAC and U.S.. More than 87% of the HF deaths in Paraiba and Brazil involved patients older than 60 years old. There was a higher frequency of woman admitted for HF, both in Paraiba and Brazil, with similar mortality rates when compared to men. Since women are generally underrepresented in clinical trials, there is a need for more studies focusing on that population. Hospital-based clinical studies should be performed to identify the causes for the trend of increase in in-hospital mortality rate for HF.
